# Antifungal action and targeted mechanism of Bio fabricated zinc oxide (ZnO) nanoparticles against *Ascochytafabae*

**DOI:** 10.1016/j.heliyon.2023.e19179

**Published:** 2023-08-21

**Authors:** Indu Sharma, Manu Vineet Sharma, M. Akful Haque, Jesus Simal-Gandara

**Affiliations:** a Department of Physics, Career Point University, Hamirpur, H.P., India; b Department of Botany, Career Point University, Hamirpur, H.P., India; c Department of Pharmaceutical Analysis, Anurag University, Hyderabad, Telangana, India; d Universidade de Vigo, Nutrition and Bromatology Group, Analytical Chemistry and Food Science Department, Faculty of Science, E32004, Ourense, Spain

**Keywords:** ZnO NPs, Sol gel method, XRD, UV, RAMAN analysis, *Ascochytafabae* and antifungal activity

## Abstract

The current work focuses on analysing the structural, optical, and anti-fungal efficacy of ZnO nanoparticles using well diffusion agar methods and minimum inhibitory concentration (MIC). ZnO nanoparticles were created using the sol gel method. To check the synthesized material's spatial and optical characteristics, XRD, UV, and RAMAN studies were performed. The median diameter of produced nanostructures is in the region of nanometre, according to XRD measurements. Results from Raman Spectroscopy for the nanostructure are provided, together with comparisons to current development theory and reliable experimental data. The band gap of the zinc oxide sample is found by graphing (h) 2versus input photon energy and gradually decreasing the linear component of the (h) 2 to zero. The band gap energy is expressed by the line's intersection with the energy axis. Calculations show that the energy band gap is 3.22eV.The fungus Ascochytafabae is in control of the *Phaseolus vulgaris* L. (beans) blight disease. It mostly affects the plant's stem, leaves, and fruits. *Phaseolus vulgaris* plant leaf with Ascochytafabae infection was isolated, and ZnO nanoparticle effects were observed. It emerged that the synthesized ZnO nanoparticles were highly efficient against Ascochytafabae. By using the well diffusion method and an absolute concentration of ZnO nanoparticles, the maximum inhibitory concentration was 15.0 ± 0.2 mm.

## Graphical abstract

Graphical abstract of the process of synthesis of ZnO and its characterization.

**Figure:**
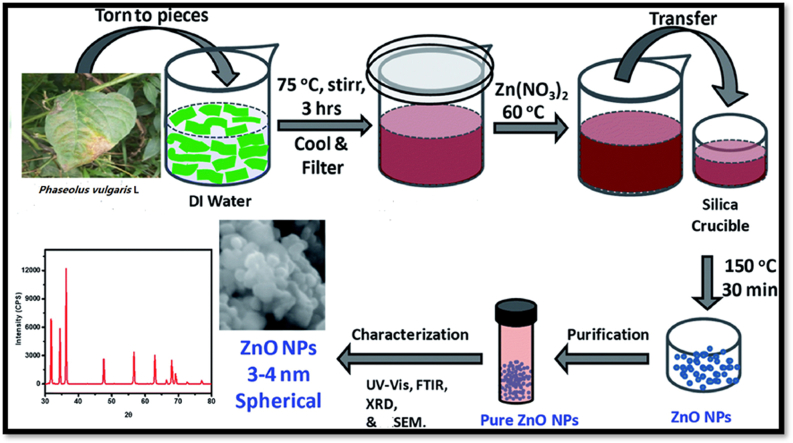

## 1 Introduction

Higher surface atom densities and higher surface reactivity are characteristics of nano-structured materials. Because of this, nano-materials have recently gained a lot of attention in both basic and applied sciences, as well as related fields [1] Inorganic materials like metals and metal oxides are more durable in many ways than organic compounds. ZnO NPs a type of metal oxide, have drawn the most attention as an anticancer, antibacterial, and antifungal substance [2]. In comparison to other transition metals like iron and copper, zinc is the most commonly used metal in biological systems because of its stable interaction, coordination flexibility, and non-toxicity [3]. Traditional chemical and physical techniques for Nano synthesis can be broadly divided into two categories: both a top-down and bottom-up strategy. In order to create nano-scale materials, the “top-down artificial strategy” for nano-materials makes use of thermal, lithographic, laser, and mechanical energy. Radiofrequency (Rf), sputtering, laser ablation, arc discharge, atomization, annealing, electron beam evaporation, and focused ion beam lithography. Atomic layer formation and chemical vapour deposition, on the other hand, are examples of the “bottom-up approach” and are gas-phase techniques, whereas metal salt reduction, sol-gel procedures, template preparation, and electrodeposition are liquid-phase techniques [4]. Wurtzite, zinc blende, and rock salt seem to be three distinct crystal formations of the inorganic semiconductor ZnO. Wurtzite has four oxygen atoms tetrahedrally bonded to each zinc atom, resulting in a structure that is thermodynamically stable at room temperature. It has multiple benefits in a wide range of industries, including semiconductors, piezoelectric devices, cosmetics, drug delivery systems, antibacterial agents, ultraviolet light (UV) shielding materials, biosensors, gas sensors, fillings in medical materials, and fillings in medical materials [5]. Because of their durability under working circumstances and reliability, many metal oxides, particularly TiO_2_, NiO, ZnO, MgO, and CuO, are being used in a diverse range of industries. According to numerous reports, amongst metal and metal oxide nanoparticles, magnesium oxide (MgO) and zinc oxide (ZnO) nanoparticles exhibit wide ranging colour deterioration and antibacterial activity. The crucial properties of MgO and ZnO, such as their excellent heat resilience, low electrical conductivity, plus catalytic action, are usually strengthened further at the nanoparticle scale [6]. Effect of ZnO Nanoparticles on Growth and Biochemical Responses of Wheat and Maize, Germination of *Triticum aestivum* Seeds, *Brassica nigra* Seedlings and Stem Explants have been studied and analyzed, and found that ZnO particles have shown prominent Growth Dynamics and Antioxidative Response [[7], [8], [9]]. Moreover, the study of ZnO nanoparticles in Energy, Biomedicine, and Environmental applications for Roadmap to the Future have also been made [10].

ZnO NPs can be chemically produced via a variety of processes, including spray pyrolysis, hydrothermal treatment, sol-gel, co-precipitation, combustion, and sonochemical reactions, among others [2]. Many researchers have made attempts to improve its ability by forming a hetero-junction with the metal oxides or by doping of other ion as impurities. To understand the antifungal activity of ZnO based nanoparticles along with the detailed mechanism the morphological analysis using microscopic instrument is believed to play important role. Various microscopic techniques like SEM, TEM gives the detailed information. But here we have the synthesized ZnO NPs by using sol-gel method and studied structural and optical properties. The anti-fungal properties of ZnO NPs have also been studied by using agar-well diffusion method against the phytopathogen *Ascochytafabae* with PDA medium.

## 2 Materials and method

### 2.1 Materials

Fresh *Phaseolus vugaris* plant (Voucher no: AUBH10866) was collected from Thiruvananthapuram, Kerala (8.5241 N, 76.9366 E) and identified by a taxonomist at the Department of Botany, University of Tirupathi. Synthesizing Zinc Oxide nanoparticles via sol gel technique in this research includes the use of several materials such as Zinc Acetate Dihydrate (Zn(CH3COO)2.2H2O) ≥99% purity (HmbG Chemicals), Sodium hydroxide (NaOH) ≥98% (Sigma Aldrich), Ethanol (CH2COOH) HmbG Chemicals) and distilled water. Zinc Acetate Dihydrate was used as precursor and Ethanol was used as a reagent. Distilled water was used as a solvent medium.

### 2.2 Preparation of the materials

Sol gel technique was used to create zinc oxide nano-particles. In a 500 ml beaker of ethylene glycol, a 12:1 M solution of C_4_H_12_O_6_Zn, NaOH was produced. At 80 °C, these mixtures were continuously swirled for 30 min. Drop by drop, aqueous organic solvent was then incorporated into the reaction mix to get the pH level to 9.0. To create a uniform solution, the process was continually agitated for 3 h. Last but not least, the material was calcined for 3 h at 500 °C to produce a fine powder of zinc oxide nanoparticles [11].

### 2.3 Isolation of fungi

The fungi were isolated from the infected leaves of *Phaseolus vulgaris* L. plant. The collection of infected leaf was based on the selection of morphological features. Samples were brought to laboratory at 4 °C temperature after collection from crop fields of Shivalik Zone of Western Himalaya. Tissue from the infected leaf was inoculated on the PDA medium under sterile conditions and Chloramphenicol was used as control agent for the growth of bacteria. The tissue was incubated in Petri dish at 27 °C for a week in dark conditions [12,13]. The obtained culture was further subculture through single spore isolation method in triplicate to get pure culture [14].

### 2.4 Identification of fungi

Preliminary identification of the phytopathogenic fungal strain was carried out on the basis of morphological characters of colony [15], fruiting bodies and spores [12,15]. The culture was transferred to the National Center of Fungal Taxonomy in New Delhi for final identification and accession number was obtained.

Electron Microscopical Studies: This technique was used mainly to determine the size and morphology of the particles, as well as their state of agglomeration, to discover the effect of the different synthesis parameters considered on the characteristics of the final product. To obtain the micrographs with Scanning Electron Microscopy (SEM), the synthesized solids were suspended in 1 ml of ethanol and this suspension was placed in an ultrasonic bath for 1 h. A small amount was subsequently taken using a Pasteur pipette and deposited on a nickel grid previously covered with a Formvar membrane. The grid was placed in the sample holder of the Jeol Model JSM 7100F electron microscope equipped with the Oxford EDS detector was used and the sample was observed.

### 2.5 Antifungal activity of ZnO nanoparticles

The antifungal activity of ethanol extract of ZnO nanoparticles was calculated against isolated phytopathogenic fungi by well diffusion method. The 1 ml of fresh fungal culture was pipetted out in sterile petri plate and sterile PDA medium was poured in the plate under sterile conditions. After solidification, four wells of 6 mm diameter each were contrived with the help of sterile cork borer in agar plate containing inoculum. These wells were labelled as A, B, C and D. Four different concentrations of stock solution (15 μg/ml) were prepared by serial dilutions i.e., 25%, 50%, 75% and 100% Well A was loaded with 25%, Well B was loaded with 50%, Well C was loaded with 75% and well D was loaded with 100%. In whole this experiment Griseofulvin (15 μg/ml) was used as positive control agent and ethanol was used as negative control. The plates were incubated for a week at 27 °C under dark conditions. After a week the zones of inhibition around the well were recorded to determine the Minimum Inhibitory Concentration. The mean value of triplicate values was considered. Whole the experiment was carried out in triplicate. The data was subjected for statistical analysis by using origin pro-7.6.

## 3 Result and discussion

### 3.1 X-ray diffraction studies

**Fig. 1:**
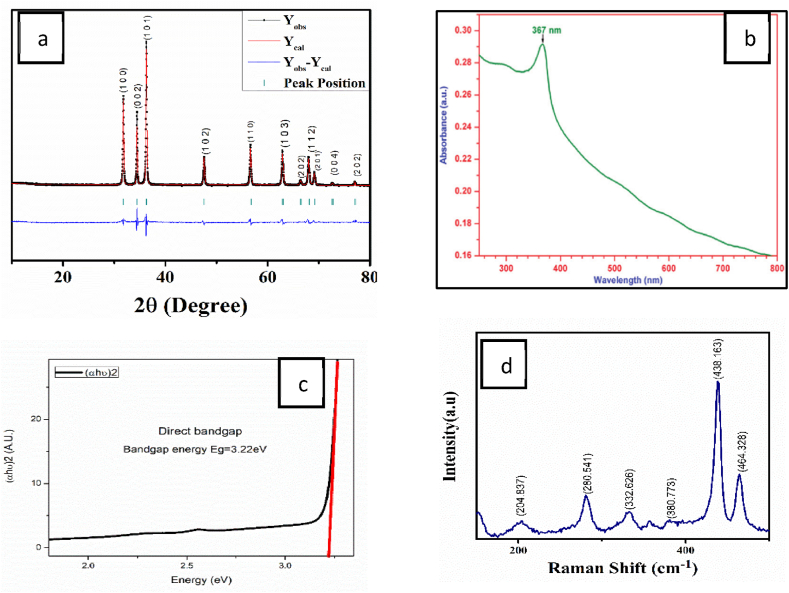
Characterization of the ZnO-Nps a) The diffraction peaks Rietveld refinement for the sample ZnO, b) UV–vis spectrum of ZnO, c) Representation of band gap using photon energy d) Raman Spectroscopy Analysis of ZnO NPs.

The Fullproof Suite software was used to examine the XRD pattern using the Rietveld method using space group *P*6_3_*mc* [15]. The X-ray diffractogram obtained from Rietveld-refined data are illustrated and shown in Fig. 1(a). It is clear from Fig. 1(a) there are no peaks of impurity because of that the high purity ZnO was successfully obtained. The Debye-Scherrer formula considers β as peak broadening of the half-maximum, the λ gives the wavelength of X-ray used, and the θ is the angle known as Bragg diffraction angle, yielding particle sizes of D = 0.94λ/β(cosθ). Using the Debye-Scherrer formula, the average crystallite size D is 42 nm. The figure illustrates different ZnO peaks that indicate the crystallinity of nature. The ZnO nano-powder, which was synthesized, identified a single phase with distinct diffraction peaks. At position 36.1897°, the highest peak from plane with a 16000-intensity value, and the second highest peak is at an angle of 31.94° in the plane with a 10000-intensity value [16]. Table 1 lists the parameter's values that have been determined during refining. The goodness of fitting (GOF) score for each sample (<3), indicating, between the computed and observed information, there is high agreement.

**Table 1:** Parameters values acquires from refinement.

a (A^0^)	c (A^0^)	c/a	Volume (A^03^)	Goldenness of fitting (GOF)
3.2487	5.2049	1.6021	54.9327	2.47

### 3.2 Ultraviolet–visible (UV–vis) spectroscopy

UV–vis spectrum of ZnO shown in Fig. 1(b). By using a Tauc plot of (αhν)^2^ versus (hν) and expanding the linear sections of the curves to the energy axis shown in equation [1], it is possible to determine the band gap energy (E_g_) of ZnO samples from the Ultra Violet - visible spectra [17].

[1] $${\left(\alpha .h\nu \right)}^{2}=A\;(h\nu -E_{g})\;$$

where, the absorption coefficient is α, the photon energy is hν, the direct band gap energy is E_g_, and constant is A. The photon energy is defined by h, the Planck constant (6.62610^−34^Js), and ν, the frequency, which is equal to c/α, where c is the light's speed in metres per second (2.99 m/s) and α is the light's wavelength [18]. Energy value for band gap is represented by the intersection point at the energy axis. The value of energy band gap is calculated to be 3.22 eV as shown in Fig. 1(c).

### 3.3 Raman Spectroscopy Analysis

Using the criterion for choosing phonon resonance modes, the A1 and E1 branches exhibit Raman and infrared activity; The E2 stems solely show Raman activation, while the B1 branches show combination of infrared and Raman inactivity. The constituents of the longitudinal optical (A1L and E1L) and transverse optical (A1T and E1T) of the phonons with A1 and E1 modes (symmetry) are polar and have distinct frequencies. It is caused by both the crystal field connected to the phonons and the long-range electrostatic forces [19]. Fig. 1(d) illustrates the Raman spectra, which range from 200 to 500 cm^−1^. The E_2_ height of ZnO, the strongest mode in the crystal structure of wurtzite, can be attributed to the finest and sharpest peak at about 438.163 cm^−1^ [[20], [21], [22], [23]].

### 3.4 Isolated and identified fungi

**Fig. 2:**
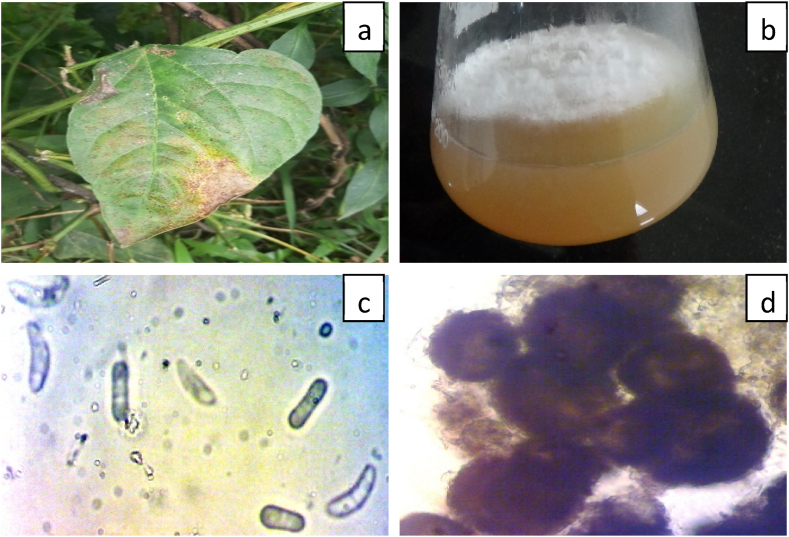
a) Leaf showing symptoms of blight diseases b) Culture of *Ascochytafabae.* c) Pycnidia of *Ascochytafabae* d) Conidia of *Ascochytafabae.*

Rust colored oblong and elliptical spots were observed on the leaves. Later on, these spots overlap to form irregular shapes. Morphologically it appears like leaf blight (Fig. 2(a)). This is a seed born disease which initially affect stem then leaves and finally fruit. Infected leaf tissue was cultured was colony was observed. Initially the colony was white and velvety but later on it changes in to ash white color with dark brown spots of conidia. The margin of the colony was entire and radial having growth rate 89.0 ± 0.5 mm from 7th day of the inoculation (Fig. 2(b)). The hyphae were smooth and septate. Nearly globose, dark brown colored pycnidia of 205–252 μm diameter were observed (Fig. 2(c)). The conidia were hyaline, straight and slightly curved with two to three septa. The basal side of the conidium was slightly rounded and size of conidium varied from 15–23 × 3.5–5.5 μm (Fig. 2(d)). The isolated fungus was *Ascochytafabae* having accession number 9856.20 provided by NCFT. This fungus is responsible for blight of *Phaseolus vulgaris* L. It is a severe Phyto disease in local fields of Western Himalaya which mostly affect leaves and fruits.

### 3.5 Morphological study of ZnO nanoparticles

**Fig. 3:**
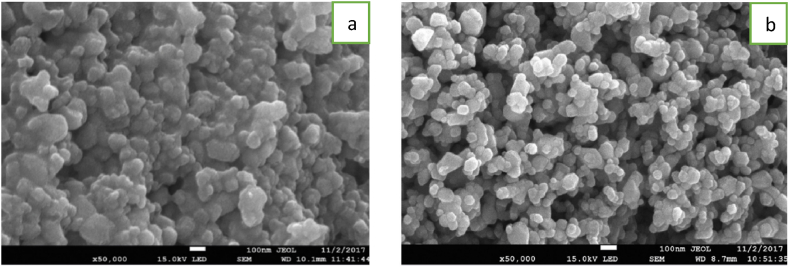
Morphological studies (SEM) images of ZnO nanoparticles at various magnifications.

Fig. 3(a and b) shows the micrographs obtained with SEM of the ZnO synthesized by a chemical route, as well as its elemental composition obtained with the EDS microprobe. In them it is observed that the particles had a spheroidal morphology and a particle size smaller than 100 nm Fig. 3(a and b) shows quite homogeneous and powders with low agglomeration. The zinc (63.62% of Zn (65.38 u) and 26.38% of O (15.999 u). These percentages indicate that the ZnO synthesized was not stoichiometric and, therefore, presented defects, mainly interstitial oxygen (Oi) given the excess of this element in the solid [24,25].

### 3.6 Antifungal activity

The synthesized ZnO nanoparticles are quite effective against this noxious phytopathogen. At different concentrations these particles have inhibited the *Ascochytafabae* differently. Following is Table 2 showing zone of inhibition against *Ascochytafabae* in mm. In this study ethanol is the negative control and griseofulvin (15 μg/ml) is the positive control. In Table 2 shows effect of ZnO nanoparticles against Ascochytafabae.

**Table 2:** Effect of ZnO nanoparticles against *Ascochytafabae.*

Sr.No.	Concentrations	Zone of Inhibition (mm)
1	25%	7.0 ± 0.2
2	50%	9.0 ± 0.2
3	75%	12.0 ± 0.2
4	100%	15.0 ± 0.2
5	Positive control	18.0 ± 0.2

**Fig. 4:**
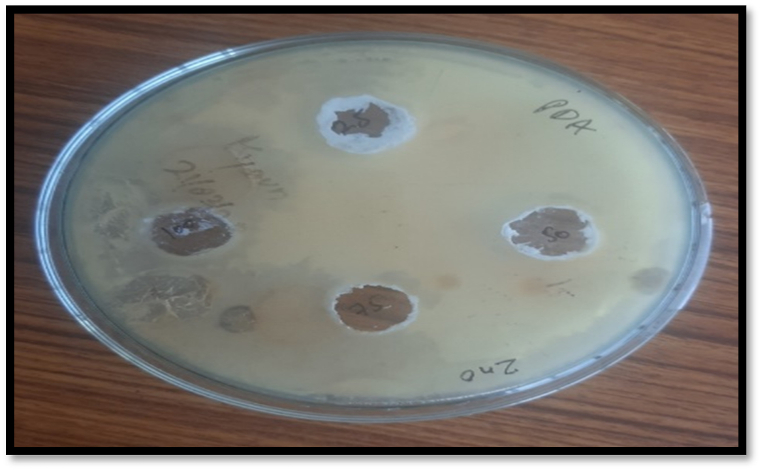
Antifungal activities of ZnO NPs.

The ethanolic extract of ZnO show maximum inhibition zone at 100% concentration (15 μg/ml) i.e., 15.0 ± 0.2 mm as compare to negative control. At absolute concentration, zone of inhibition is 3.0 ± 0.2 mm less than the inhibitory zone for positive control. The minimum inhibition zone was recorded at 25% concentration i.e., 7.0 ± 0.2 mm (Fig. 4) as compare to negative control. It is 11 ± 0.2 mm less than the inhibitory zone for positive control.

## 4 Conclusion

The current study uses well diffusion agar methods to investigate the structural, antifungal as well as optical characteristics of nanoparticles of ZnO at the minimal inhibitory dose (MIC). ZnO nanoparticles were created using the sol gel technique. The structural and optical characteristics of ZnO nanoparticles were investigated using XRD, Ultra Visible spectra, and RAMAN studies. The average crystallite size of nano-crystals is in the range of nm, according to XRD measurements. Results from Raman Spectroscopy for the nanostructure are provided, together with comparisons to current development theory and reliable experimental data. The calculated energy band gap value is 3.22 eV. The fungus that causes Phaseolus vulgaris blight is known as Ascochytafabae (beans). It mostly affects the plant's stem, leaves, and fruits. ZnO nanoparticles have an effect on Ascochytafabae, which has been isolated from a *Phaseolus vulgaris* plant leaf that has been infected. It was discovered that the synthesized ZnO nanoparticles were extremely effective against Ascochytafabae. By using the well diffusion method and an absolute concentration of ZnO nanoparticles, the maximum inhibitory concentration was 15.0 ± 0.2 mm. This study can be beneficial for the growth control of Ascochytafabae, and to detect the various type of unknown fungi by following the suggested pattern of investigation and by referring the observed data.

## Funding

Start scheme, Department of industry Govt. of Himachal Pradesh HPSTARTUP/2020/08/20. Funding for open access charge: 10.13039/501100006761Universidade de Vigo/CISUG.

## Ethical approval

Approved from all the ethical point of view.

## Author contribution statement

Indu Sharma and Manu Vineet Sharma: Contributed reagents, materials, analysis tools or data; Performed the experiments.

M. Akful Haque: Conceived and designed the experiments data and wrote the paper.

Jesus Simal-Gandara: Analyzed and interpreted the data and wrote the paper.

## Data availability statement

Data will be made available on request.

## Additional information

No additional information is available for this paper.

## Declaration of competing interest

The authors declare that they have no known competing financial interests or personal relationships that could have appeared to influence the work reported in this paper.
